# Haploid Meiosis in *Arabidopsis*: Double-Strand Breaks Are Formed and Repaired but Without Synapsis and Crossovers

**DOI:** 10.1371/journal.pone.0072431

**Published:** 2013-08-07

**Authors:** Marta Cifuentes, Maud Rivard, Lucie Pereira, Liudmila Chelysheva, Raphael Mercier

**Affiliations:** 1 INRA, UMR1318, Institut Jean-Pierre Bourgin, Versailles, France; 2 AgroParisTech, Institut Jean-Pierre Bourgin, Versailles, France; Institute of Botany, Chinese Academy of Sciences, China

## Abstract

Two hallmark features of meiosis are i) the formation of crossovers (COs) between homologs and ii) the production of genetically-unique haploid spores that will fuse to restore the somatic ploidy level upon fertilization. In this study we analysed meiosis in haploid *Arabidopsis thaliana* plants and a range of haploid mutants to understand how meiosis progresses without a homolog. Extremely low chiasma frequency and very limited synapsis occurred in wild-type haploids. The resulting univalents segregated in two uneven groups at the first division, and sister chromatids segregated to opposite poles at the second division, leading to the production of unbalanced spores. DNA double-strand breaks that initiate meiotic recombination were formed, but in half the number compared to diploid meiosis. They were repaired in a RAD51- and REC8-dependent manner, but independently of DMC1, presumably using the sister chromatid as a template. Additionally, turning meiosis into mitosis (*MiMe* genotype) in haploids resulted in the production of balanced haploid gametes and restoration of fertility. The variability of the effect on meiosis of the absence of homologous chromosomes in different organisms is then discussed.

## Introduction

Meiosis is a specialized type of cell division by which sexually reproducing eukaryotes produce new combinations of alleles in gametes. This process occurs with two successive rounds of chromosome segregation following a single replication. Crossovers (COs) (reciprocal exchange of genetic material) are formed between homologues during prophase I of meiosis [1]. Crossover formation is initiated by the formation of DNA double-strand breaks (DSBs) catalyzed by the SPO11 protein. DSBs generate single stranded DNA tails that are coated by RAD51 and DMC1 proteins to form nucleoprotein filaments. These DNA-proteins filaments are involved in active homology searches and strand exchanges, a prerequisite for the alignment of homologous chromosomes and DSB repair [2]. In meiosis, DSBs can be processed either using the sister chromatid or one of the homologous chromatid as a template, but inter-homolog repair is required to promote CO formation between homologous chromosomes. Inter-homolog repair leads to both COs, reciprocal exchange of homomogous chromatid continuity, and non-crossovers (NCOs) which are short non-reciprocal exchange between homologues [2,3]. The two homologous chromosomes thus become physically linked by chiasmata, the cytological manifestation of COs, that, in conjunction with sister chromatid cohesion, form a bivalent. Release of chromosome arm cohesion permits chromosomes to separate from their homologues and to migrate to opposite poles at the first division. Sister chromatids segregate at the second division leading to the formation of four balanced haploid meiotic products [1].

In parallel with recombination progression during prophase I, homologous chromosomes pair along their length *via* a tripartite ladder-like proteinaceous structure called the synaptonemal complex (SC) [4]. The SC is composed of the two axial elements of the homologous chromosome that were formed at leptotene, and the central element which polymerizes between the two axial elements (then called the lateral elements) holding homologous chromosomes together in a process completed by pachytene. In *Arabidopsis* ASY1 and ASY3 are components of the axial element [5,6] and ZYP1a and ZYP1b are proteins of the central element of the SC [7].

*Synapsis* and recombination are two inter-related processes. In many organisms including *S. cerevisiae* and *A. thaliana*, synapsis is dependent on DSBs and strand invasion [4,8]. However, the relationship between COs, which is one of the products of recombination, and synapsis is more complex. *Synapsis* can occur in *Arabidopsis* even if CO are not eventually formed, as shown in the *zmm* series of mutants [9,10]. In contrast synapsis does not occur in the corresponding *S. cerevisiae* mutants [11]. Conversely, the depletion of the central element of the SC does reduce but does not abolish CO formation in both *S. cerevisiae* and plants [7,11,12]. Thus CO formation and synapsis appear to be functionally related, but not firmly inter-dependent.

DSB repair using the homologue and homologous chromosome synapsis are two hallmarks of meiosis. However, in certain situations the homologous sequence may not be available. This is the case when DSBs occur in an insertion/deletion polymorphism [13] or in the extreme case when the homologous chromosome is not present [14]. Such a situation occurs in haploids. Indeed, haploid organisms contain only one copy of each chromosome (e.g. 5 chromosomes in *Arabidopsis* ), contrasting from a diploid which has two copies of each chromosome (e.g. 10 chromosomes in *Arabidopsis* ). Haploid plants can be produced through gametic embryogenesis or from crosses, in which one parental genome is eliminated after fertilization. The resulting haploid plants develop essentially normally and produce sexual organs in which meiosis occurs [15–17]. In some contexts meiosis can also be induced in haploid yeast. This is the case under conditions that mimic the situation of a diploid zygote such as co-expression of mating types which activates different meiotic genes in *S. cerevisiae* and *S. pombe* [18,19]. Haploidy raises a challenge for the meiotic cells because the absence of a homologue *de facto* prevents homologous synapsis and homologous recombination.

In budding yeast haploid meiosis, DSBs are generated and repaired [20,21] and synaptonemal complexes can elongate between non-homologous chromosomes and within single chromosomes, presumably between sister chromatids, depending on the strains [22,23]. Also, ectopic recombination has been reported [24]. Fission yeast haploids also generate DSBs that are repaired [19]. In haploid plants, the rule seems to be extensive synapsis and low chiasma frequency between non-homologous regions/chromosomes. This is the case in barley [25,26], some diploid *Brassica* species [27,28], rye [29] and rice [30]. The number of chiasma can rise in allohaploids, (haploids derived from allopolyploid plants), potentially because the chromosomes inherited from their progenitors (homeologous chromosomes) have retained a much higher level of similarity than the non-homologous chromosomes found in diploids. This is the case in allopolyploid wheat species [31,32] or oilseed rape [17,33]. It should be noted that in these allopolyploid species the number of homeologous COs that occur in haploids has been shown to be genetically controlled [34].

*Arabidopsis thaliana* is the most widely studied plant so far thanks to the available genetic tools and resources, and meiosis has been extensively described and deciphered [8,35,36]. Recently, the production of haploid *Arabidopsis* plants has been made possible thanks to the use of inducer lines [16,37]. However, meiosis in haploids has not yet been comprehensively described. The aim of this study was to analyze meiotic behavior and dynamics in the absence of a homolog in *Arabidopsis* , notably in terms of DSB production and repair, SC axial and central element formation and crossover occurrence. For this purpose, we analyzed meiosis in wild-type haploid *Arabidopsis* plants and in a range of haploid meiotic mutants.

## Results

### Synapsis and crossover formation are impaired in haploid meiosis

**Figure 1:**
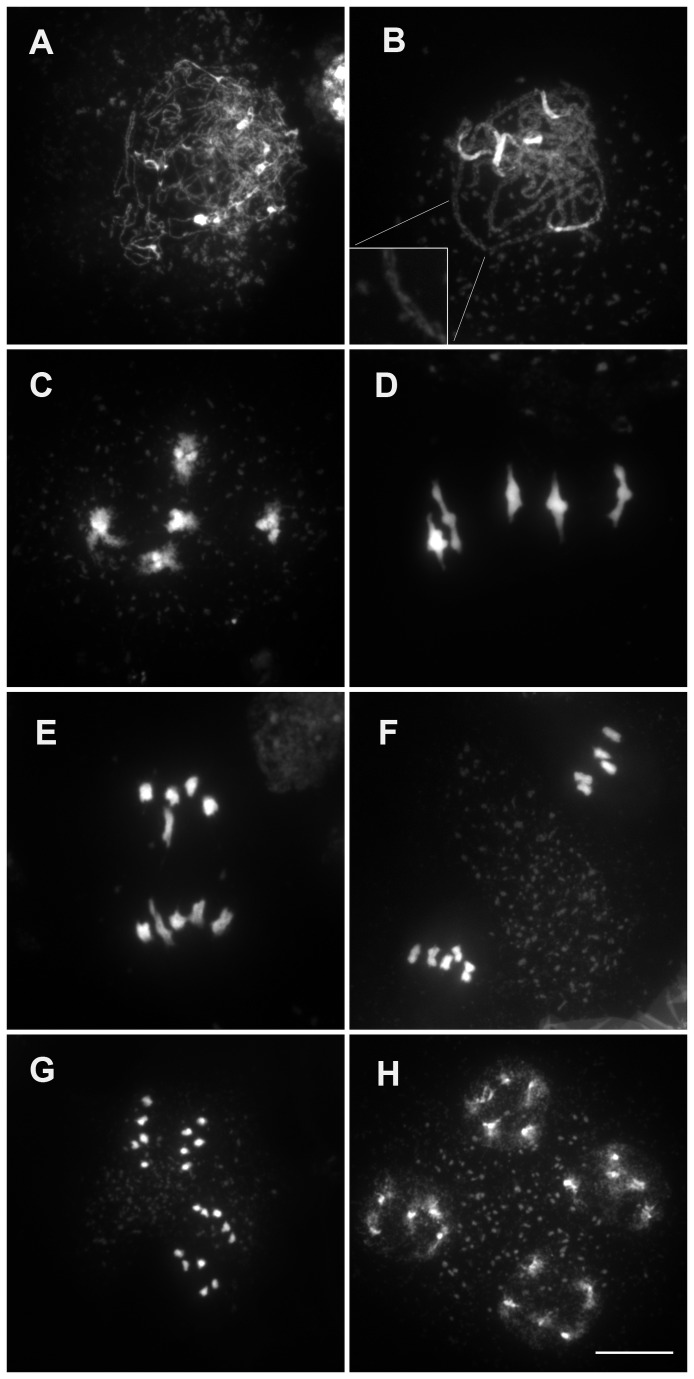
DAPI Staining of Wild-Type (Col-0) male meiocytes during meiosis. (A) Leptotene, (B) pachytene, (C) diakinesis, (D) metaphase I, (E) end of anaphase I, (F) metaphase II, (G) anaphase II, (H) end of meiosis. Bar, 10µm.

**Figure 2:**
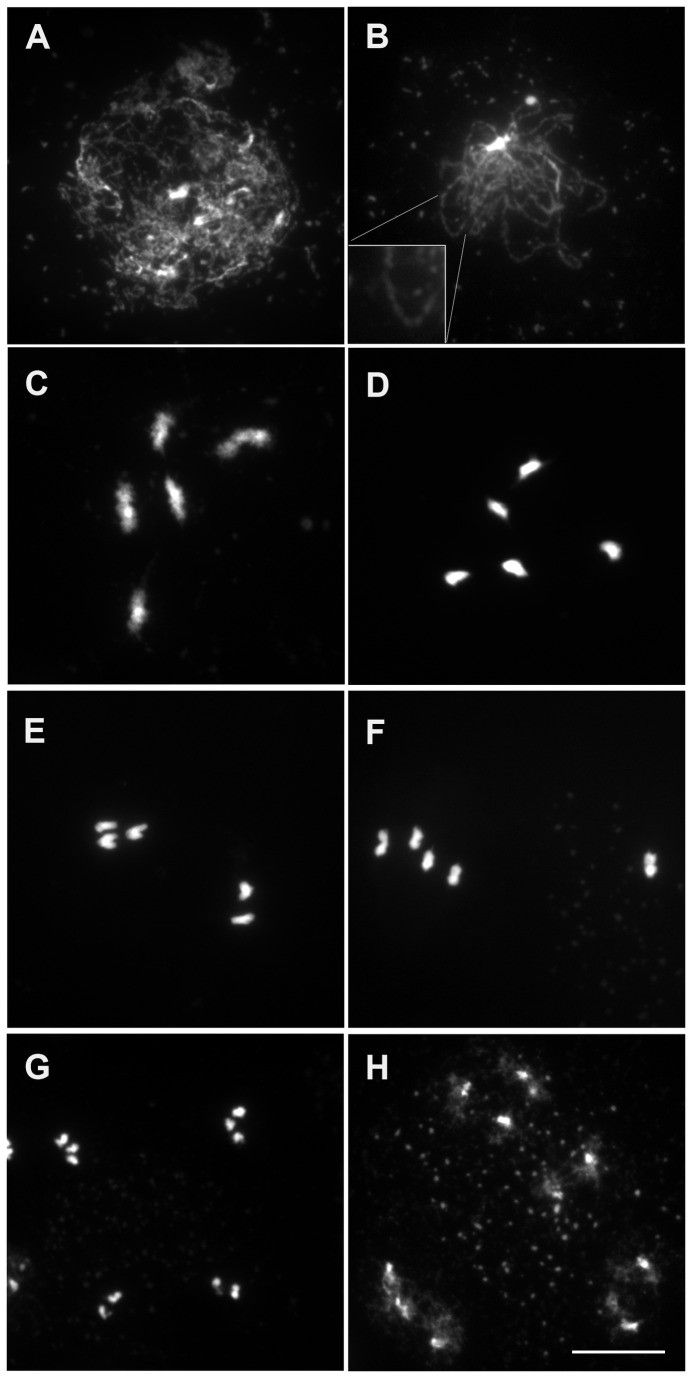
DAPI Staining of haploid *Arabidopsis* male meiocytes during meiosis. (A) Leptotene, (B) zygotene, (C) diakinesis, (D) metaphase I, (E) anaphase I, (F) metaphase II, (G) end of anaphase II, (H) end of meiosis. Bar, 10 µm.

We investigated male *Arabidopsis* haploid meiosis by observing spread meiotic chromosomes from male meiocytes. Wild-type diploid *Arabidopsis* meiosis has been described in detail [38] and Figure 1 summarizes its major stages. At leptotene, chromosomes appear as abundant tenuous chromosome threads (Figure 1A). *Synapsis* begins at zygotene and is complete by pachytene where homogous chromosomes are associated entirely along their length (Figure 1B). At diplotene, the SC disassembles and homologs are linked by chiasmata which become visible at diakinesis (Figure 1C). At metaphase I the five bivalents are maximally condensed and align on the metaphase plate with homologous centromeres directed towards opposite poles (Figure 1D). At anaphase I, homologous chromosomes migrate to the opposite poles (Figure 1E). At metaphase II individual chromosomes align on the metaphase II plates (Figure 1F) and pairs of chromatids separate at anaphase II (Figure 1G) which gives rise to tetrads of four microspores with a chromosome content n=5 (Figure 1H). In haploid male meiotic cells the chromosome threads at leptotene were indistinguishable from the diploid (Figure 2). As meiosis progresses no synapsis was detected (compare Figure 2B to Figure 1B). At diakinesis and metaphase I stages, five univalents were clearly distinguished in almost all cells analyzed (Figure 2C and 2D). Among 120 cells, one structure resembling a bivalent with one chiasma was observed. The frequency of meiotic CO in haploid *Arabidopsis* is thus extremely low (0.01/cell; n=120) compared to diploid wild type (10/cell [39]). At anaphase I the five univalents segregated in two groups, without separation of sister chromatids in 75% of the cases (n=38, Figure 2E). In the 25% of remaining cells some premature sister chromatid segregation was observed. This is reminiscent of the behavior of univalents in diploid mutants that lack recombination (e.g *spo11-1* or its partners [40,41]), except that five univalents segregate instead of ten. Consequently, instead of the two pools of five chromosomes observed at metaphase II in wild-type diploid cells (Figure 1F), variable partitioning of the five chromosomes was observed (Figure 2F). Pairs of sister chromatids segregated evenly at anaphase II (e.g 3-3 and 2-2). In some cells chromatids segregated unevenly at anaphase II presumably due to their premature separation at the first division. Unbalanced tetrads were thus produced (Figure 2G-H). As a result, the haploid plants had drastically reduced fertility compared to wild type (Table 1), but still produced some seeds, likely through the random 5-0 or 0-5 segregations (which would occur at a low rate of twice in thirty two meiosis) [16].

**Table 1:** Seeds per fruit in *Arabidopsis* haploids.

	**Haploids**									
	wt	*rec8*	*osd1*	*spo11*	*rad51*	*dmc1*	*rec8/osd1*	*rec8/spo11*	*osd1/spo11*	*MiMe*
n fruits	12	13	16	16	12	19	16	16	16	11
mean seeds/fruit	0.5	0.0	0.1	0.7	0.0	0.1	0.1	0.1	0.0	18.7

**Figure 3:**
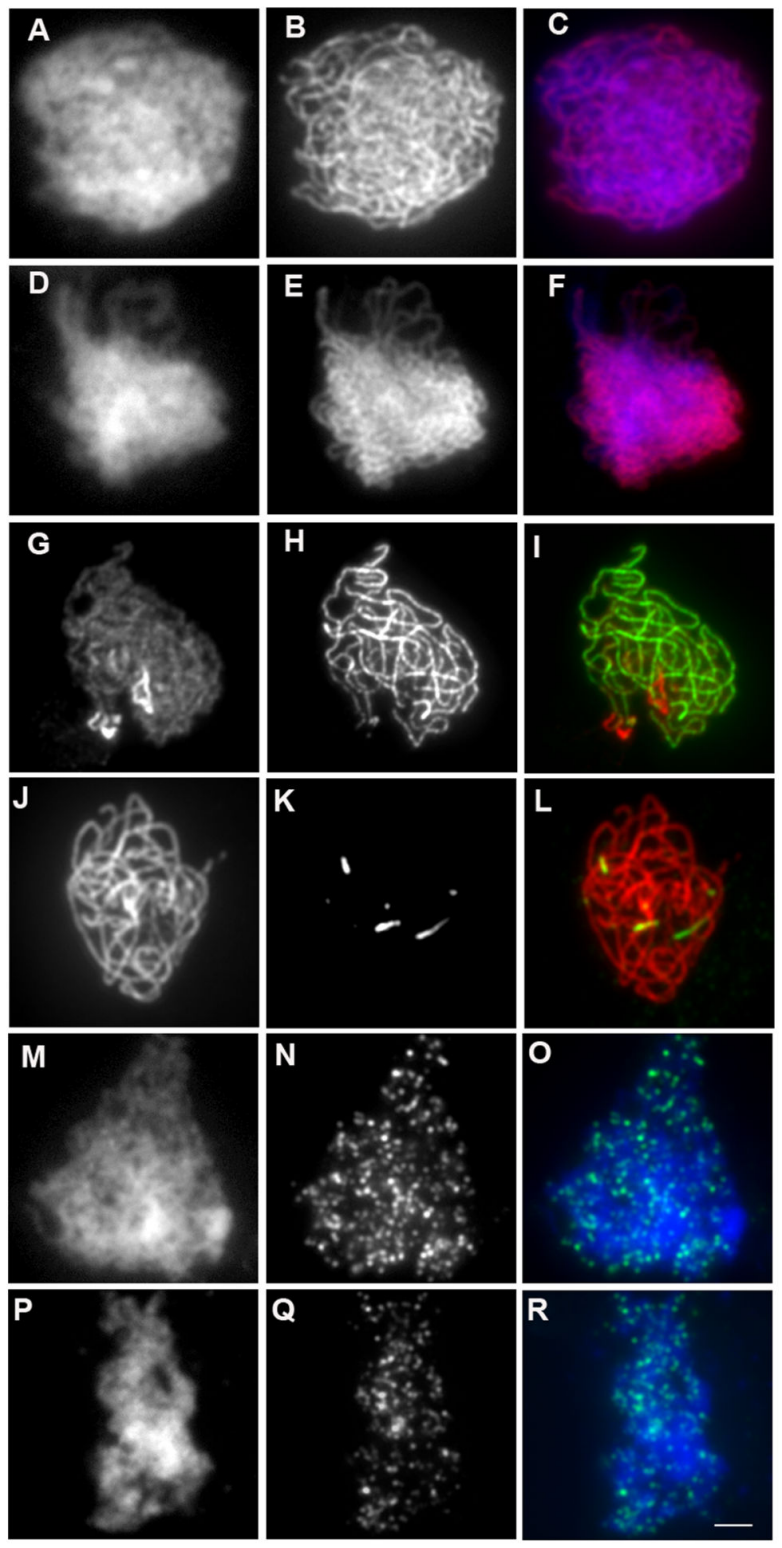
Immunolocalization of ASY1, ZYP1 and DMC1 in wild-type and haploid prophase male meiocytes. Immunolocalization of ASY1 in wild-type zygotene. A) DAPI, B) ASY1, C) merge (DAPI in blue, ASY1 in red). Immunolocalization of ASY1 in haploid zygotene. D) DAPI, E) ASY1, F) merge (DAPI in blue, ASY1 in red) Immunolocalization of ASY1 and ZYP1 in wild-type pachytene G) ASY1, H) ZYP1, I) merge (ASY1 in red, ZYP1 in green). ASY1 and ZYP1 in haploid pachytene-like. J) ASY1, K) ZYP1, L) merge (ASY1 in red, ZYP1 in green). Immunolocalization of DMC1 in wild-type meiocytes. M) DAPI, N) DMC1, O) merge (DAPI in blue, DMC1 in green). Immunolocalization of DMC1 in haploid meiocytes. P) DAPI, Q) DMC1, R) merge (DAPI in blue, DMC1 in green). Bar, 2 µm.

To more accurately analyze synapsis in haploids, meiotic chromosomes were immuno-labeled with antibodies raised against ASY1 and ZYP1. In diploids, ASY1 labelling delineates chromosome axes with a continuous signal during leptotene and zygotene [5] while ZYP1 appears at zygotene and elongates yielding a mixture of foci and short stretches. The number of these first ZYP1 sites varies from one to more than 20 [42]. By pachytene, ZYP1 fluorescent signals extended the entire length of the five fully synapsed homolog pairs (Figure 3I) [7]. In haploids, meiotic axial elements appear similar to those of diploids, with ASY1 immunolabelling showing strong continuous staining (Figure 3F). In contrast, we never observed fully extended synaptonemal complexes in haploid meiocytes. Only a few stretches of ZYP1 labelling were observed (4.2±2.9/cell) and the total length of the SC represented on average 2% of the length of SC in wild-type diploids (154±31µm [42], *versus* 3.6±2.4µm in haploids, n=74) (Figure 3L).

**Figure 4:**
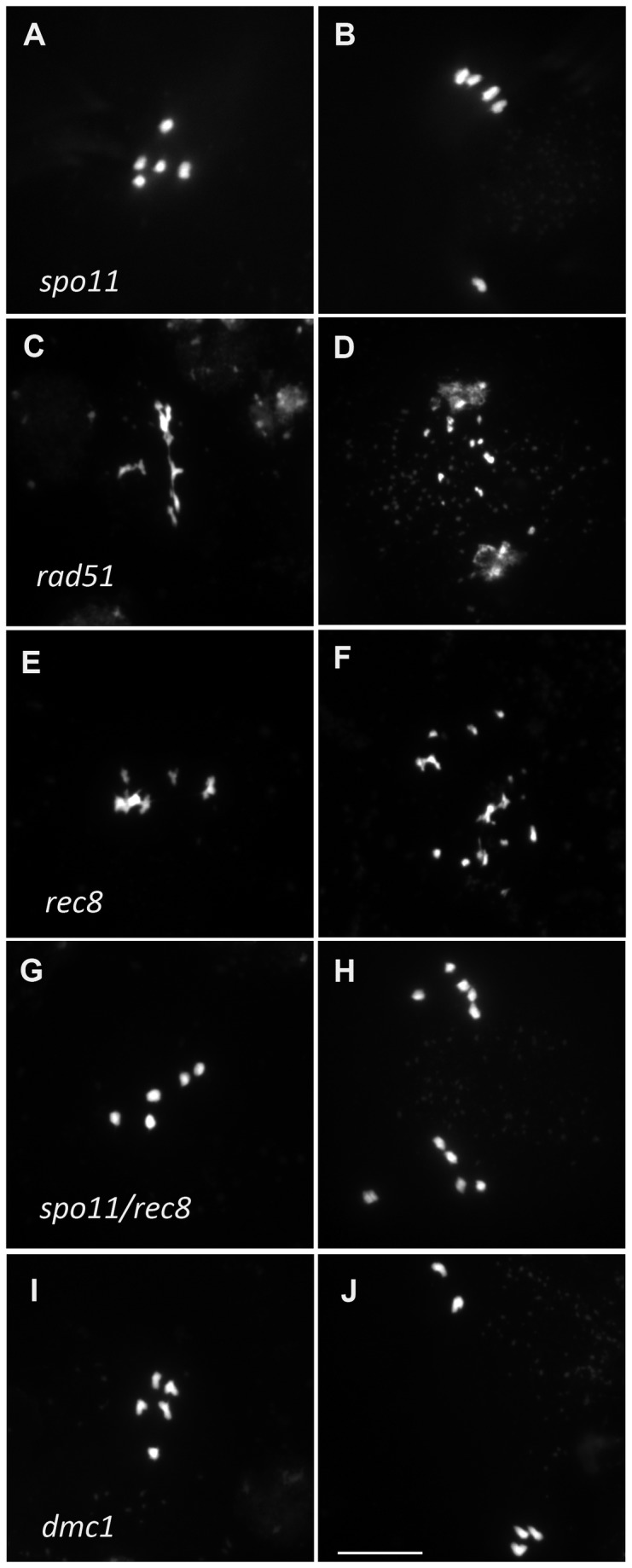
DAPI staining of male haploid meiocytes at metaphase I-anaphase I transition and during anaphase I. Several genotypes are shown: *Atspo11-1-1* (A, B), *Atrad51* (C,D) *Atrec8* (E,F) double mutants *Atspo11-1-1 Atrec8* (G-H) and *Atdmc1* (I,J) at metaphase I-anaphase I transition (left-hand column) and during anaphase I (right-hand column). Bar, 10 µm.

To test whether the residual stretches of ZYP1 in haploids were dependent on DSB formation, we analyzed meiotic progression in *Atspo11-1* haploids. Chromosome behavior in the *Atspo11-1* haploid meiosis, based on DAPI staining, showed no detectable differences from the wild-type haploid, both being similar to the diploid *Atspo11-1* apart from the number of chromosomes participating in meiosis (Figure 4A). ZYP1 immulolocalization in the *Atspo11-1* haploid, revealed, as in the wild-type haploid, very limited extent of synapsis (3.3±1.9 stretches per cell, total length 3±2 µm on average), showing that the short stretches of ZYP1 detected in wild-type haploid do not depend on programmed DSB.

### DSBs are formed and repaired in haploids

The very limited COs and synapsis in the haploid raised the question of formation of DSBs in this context. The RAD51 recombinase and the REC8 cohesin are both essential for meiotic DSB repair, with their depletion leading to *spo11*-dependent chromosome fragmentation at meiosis in the diploid [43–46]. To test whether the DSBs were formed in the haploid, we analysed haploid *rad51* and *rec8* mutants. In both *rad51* and *rec8* haploids, chromosomes showed aberrant structures at metaphase I and extensive fragmentation at anaphase I (Figure 4C-F), like in their *rad51* or *rec8* diploid mutant counterparts. The fragmentation in *rec8* was abolished by the *spo11-1* mutation (Figure 4H). This strongly suggests that DSBs are formed in the haploid, but are repaired in a RAD51- and REC8-dependent manner.

Beyond RAD51, another recombinase, named DMC1, is required for crossover formation, but not essential for DSB repair in diploid *Arabidopsis* meiosis [47,48]. DMC1 immuno-staining during meiotic prophase is thought to mark DSB sites on meiotic chromosomes. In wild-type diploid meiosis, DMC1 forms about 235 foci per cell (235±84, n=43 [42]). In wild-type haploid meiosis, DMC1 formed well defined foci, similar to those seen in diploids (Figure 3N, 3Q). However, the mean number of DMC1 foci was 121±30 (n=60), almost exactly half the amount of foci found in the diploid. This further supports that DSBs are formed in haploid meiocytes, but in half the number of wild type. In diploid *dmc1* mutants, DSBs are formed and repaired in a RAD51-dependent manner with neither synapsis nor CO formation [47,48], presumably using the sister chromatid as a template. We thus wondered if DMC1 is dispensable for the repair of DSBs in the haploid. In *dmc1* haploids, meiosis was indistinguishable from wild-type haploids (Figure 4I, 4J), and notably did not show the chromosome fragmentation that occurs in *rad51* haploids, showing that DMC1 is not essential for meiotic DSB repair in the haploid.

**Figure 5:**
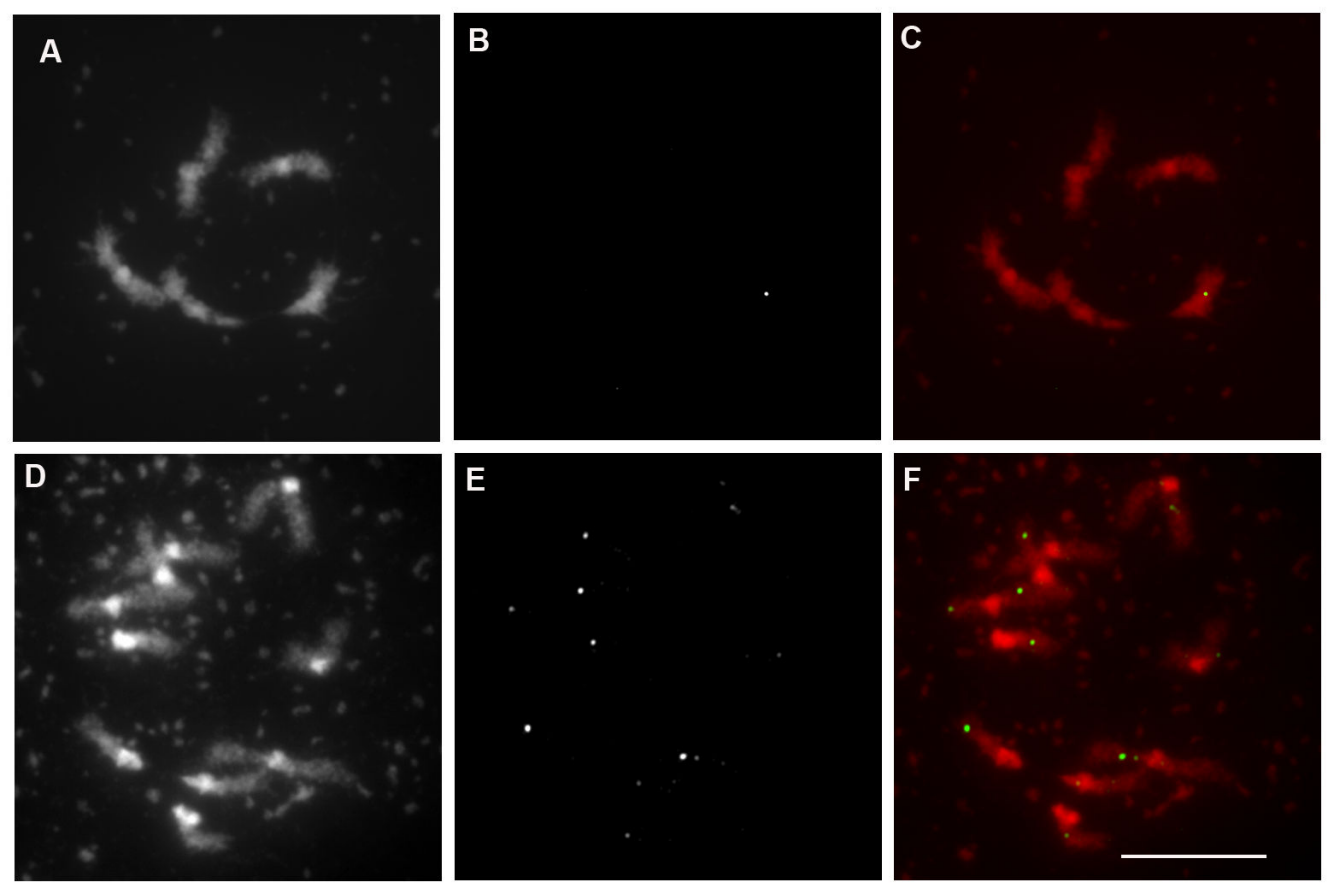
Immunolocalization of MLH1 in wild-type haploid and *Atdmc1* meiocytes. A) DAPI staining of haploid diakinesis. B) Immunolocalization of MLH1 in wild-type haploid. C) Merge of A (in red) and B (in green). D) DAPI staining of *Atdmc1* diakinesis. E) Immunolocalization of MLH1 in *Atdmc1*. F) Merge of D (in red) and E (in green). Bar, 10 µm.

MLH1 foci mark the sites of class I COs at diakinesis where it colocalises with chiasmata in *Arabidopsis* . The mean MLH1 foci number per cell at diakinesis is 9.9 for diploid *Arabidopsis* ecotype Col-0 [39]. We observed a mean of 1.6±1.4 (n=45) MLH1 foci on the univalents during diakinesis in haploid. Similarly, we observed MLH1 foci on univalents in diploid *Atdmc1* mutants with an average of 7.8 ±3 foci per cell (n=53, Figure 5).

### MiMe haploids are fertile and generate homogeneous populations of diploid MiMe plants

We previously showed that meiosis could be replaced by a mitotic-like division in *Arabidopsis* . This is achieved by combining three mutations, *spo11-1* which abolishes recombination, *rec8* which in the *spo11-1* context leads to sister chromatid separation at anaphase I, and *osd1* which provokes exit from meiosis before meiosis II [49]. This genotype called *MiMe* (for Mitosis instead of Meiosis) thus produces diploid clonal gametes, leading to the doubling of ploidy in each successive generation. With the aim of testing the effect on the *MiMe* genotype in the haploid, we analyzed each single, double and triple mutant combination.

**Figure 6:**
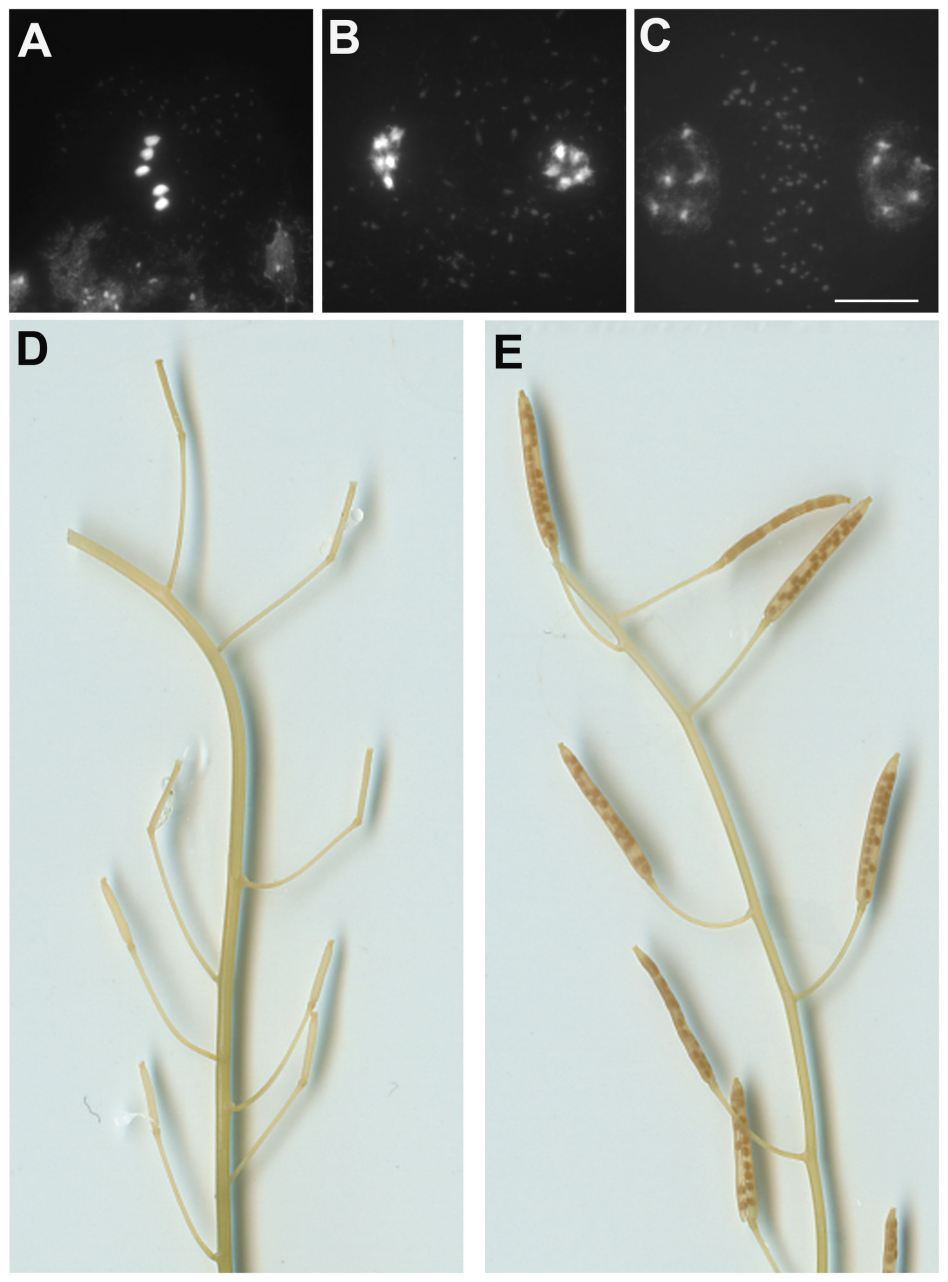
*MiMe* haploid turns meiosis into mitosis and restores haploid fertility. A) Metaphase-I, B) end of first division, C) dyad at the end of meiosis. D) Empty siliques in the haploid plant. E) Restoration of fertility in *MiMe* haploid. Bar, 10 µm.

As shown above, *rec8* mutation provoked chromosome fragmentation that was abolished by *spo11-1*. Furthermore, at anaphase I in the *spo11-1/rec8* double mutant, the five univalents where not distributed into two groups of 1 to 5 univalents (e.g 3-2) as seen in wild-type and *spo11-1* haploid cells, but instead sister chromatids segregated into two balanced groups of five chromatids (Figure 4G, 4H). This shows that like in the diploid, REC8 is essential for sister chromatid cohesion and kinetochore orientation at meiosis I. Meiosis in haploid *osd1* showed normal meiosis I, but absence of meiosis II, showing that OSD1 is required to prevent exit from meiosis before meiosis II in both diploid and haploid meiosis. The same absence of meiosis II was observed in *spo11-1/osd1* or *rec8/osd1* (not shown). In haploid *spo11-1/rec8/osd1* (*MiMe*), we observed the same behavior as in diploid *MiMe*: univalents aligned at metaphase I and segregated into two genetically identical groups of five chromatids at anaphase I without a subsequent second meiotic division, leading to the production of balanced haploid spores (Figure 6). Remarkably, while all the other genotypes produced less than one seed per fruit, haploid *MiMe* produced almost 20 seeds per fruits (Table 1, Figure 6E). These seeds developed into a homogeneous population of diploid *MiMe* plants (n=21). Thus turning meiosis into mitosis in haploid restored the fertility of haploid plants through the production of balanced clonal haploid gametes.

## Discussion

Here we showed that haploid meiosis in *Arabidopsis* proceeds without synapsis nor chiasmata. This behavior is similar to achiasmatic and asynaptic diploid mutants (e.g. *Atspo11* or *Atdmc1* [40,47]). As already shown with mutants, meiosis in plants proceed even if major defects appear, suggesting the absence of checkpoint or the possibility to overtaking them. Achiasmatic chromosomes segregate erratically at the first meiotic division and sister chromatids separate to opposite poles at the second meiotic division. Consequently, unbalanced spores are produced leading to almost total sterility. Furthermore just as meiosis can be replaced by a mitotic-like division in a diploid, the same is true for a haploid. In a haploid, *MiMe* restores the production of viable spores and in turn massively increases the fertility of the haploid by a factor of more than 30 times. This demonstrates that haploidy does not notably modify the meiotic program with the exception of recombination and synapsis dynamics.

### DSBs form in haploids

Meiotic recombination starts with formation of DSBs, which are mostly repaired using the homologous chromosome as a template [13]. As interaction between homologous chromosomes can precede DSB formation [50], we wondered if there could be a dependency on the presence of a homologous chromosome to make DSBs at meiosis. The occurrence of DSBs in haploid meiosis in budding yeast and fission yeast (de Massy et al. 1994; Callender and Hollingsworth 2010; Josef Loidl et al. 1991; Wagstaff et al. 1982; Cervantes et al. 2000) show that the presence of homologue is not a requirement for the initiation of meiotic recombination in those fungi. The occurrence of DSBs in haploid plants meiosis has not been previously established, but the presence of some chiasmata in haploid rye, barley, rice, *B. oleracea* and allohaploids derived from oilseed rape and crop wheat [17,31] are evidences for the occurrence of at least a certain level of DSB formation in those haploids. Here we showed that in *Arabidopsis* DSBs are formed in haploid meiosis. Indeed, the depletion of RAD51 or REC8 which are required for DSB repair, leads to chromosome fragmentation, which is SPO11-1-dependent (Figure 4). In addition, DMC1 foci which mark DSB repair sites [51], are formed in the haploid. Thus, the occurrence of DSBs in haploid meiosis appears to be quite universal. Remarkably, the number of DMC1 foci is half the number than in diploid meiosis, strongly suggesting that the number of DSBs is reduced by two in the haploids. The number of DSBs appears to be correlated to the amount of DNA in the cell.

### Synapsis in haploids

*Synapsis* occurs in haploids of many species. Haploid budding yeast shows extensive elongation of the SC that can involve more than 50% of the chromosome complement [22,23]. Haploids from some plants show a similar pachytene pattern. Rye and barley haploids show up to 60% and 76% of the chromosome complement involved in synapsis, respectively [25,29]. Also in rice, Gong et al. (2011) have shown that non-homologous chromosomes can form a SC in the haploid. ZEP1 (the ZYP1 homologue in rice [12] marked the entire complement in 15% of pachytene cells analysed. The presence of synaptonemal complexes in haploids of yeast and rye, barley and rice haploids indicates that in these organisms synapsis can occur with non-homologous chromosomes. In clear contrast, synapsis is abolished in haploid *Arabidopsis* (Figure 3). DSBs that are essential for synapsis in diploid *Arabidopsis* [51] are formed in large numbers in the haploid. Thus, the absence of synapsis in haploid *Arabidopsis* cannot be explained by a fault in DSB formation. What then makes *Arabidopsis* different from these other organisms? In haploid rye, whose genome is rich in heterochromatin, it has been suggested that duplicated genes in non-allelic heterochromatic regions and families of repetitive DNA could enhance interactions which will produce synapsis [29]. In haploid rice, the presence of a very similar duplicated segment in two chromosomes may explain some extent of synapsis, but not of the entire complement. Even if both rice and *Arabidopsis* have a relatively low percentage of repetitive sequence, the higher content of repetitive DNA in rice (30% of rice genome are transposable elements *versus* 10% in *A. thaliana*) [52,53] could explain the difference in haploid synapsis. Alternatively, a genetic control may prevent non-allelic synapsis in *Arabidopsis* haploids. In budding yeast, it seems that the control of synapsis in haploids could be different between strains, which may result in non-homologous synapsis [23] or the formation of ZIP1 polycomplexes within sister chromatids [22]. When comparing haploids from a range of species it seems that both genomic structure and genetic controls likely influence the dynamics of synapsis.

### Crossover formation in haploid meiosis

*Arabidopsis* haploids showed an extremely low rate of bivalents (0.01/cell). Very few chiasmata have also been detected in haploids from several other plant species. Haploids from *Brassica oleracea* present 0.14 bivalents per cell [27]. In the case of rice, frequency of chiasmata per cell is 0.25, but the only two chromosomes involved in bivalent formation in the haploid are chromosomes 11 and 12, which notably have recent gene duplications [54] and several reciprocal exchanges of chromosomal segments [55]. Rye haploids form a slightly higher frequency of 0.4 chiasmata/cell, maybe because of the abundance of non-allelic repeated blocks of DNA prone to recombine [29]. In contrast, CO frequency can be much higher in haploids derived from allopolyploid species. Chiasmata per meiosis can reach 11/cell in haploid oilseed rape [33] and 2.3/cell in wheat [31]. This is likely because homeologous chromosomes share a much higher similarity than two non-homologous chromosomes. However, there is natural variation for the number of COs in these haploids derived from allopolyploids which is clearly under genetic control [33,56]. Thus COs occur in haploids but only if sufficiently similar chromosomes are present in a favorable background.

### A bias against forming CO with the sister

In haploid *Arabidopsis* meiosis, DSBs are formed and are repaired without neither synapsis nor CO formation. Thus, repair using the sister chromatid as a template likely becomes the dominant mechanism. We showed that this repair occurs in a RAD51 and REC8-dependent manner, but independently of the presence of DMC1. This is reminiscent of the phenotype of a diploid *Atdmc1* mutant whereby DSBs are also repaired in a RAD51-dependent manner without COs and synapsis, most likely using the sister chromatid as template [47,48]. One can wonder if repair on the sister may generate sister COs in these contexts. MLH1 foci mark homologous crossovers in diploid *Arabidopsis* , unambiguously co-localizing with chiasmata sites [39]. In haploids we show the presence of 1.6 MLH1 foci per cell, which were localized on univalents (pairs of sisters without chiasmata). Similarly, around 8 MLH1 foci per cell were detected on univalents in diploid *Atdmc1* mutants (7.8 ±3, n=53, Figure 5). This suggests that COs marked by MLH1 are formed between sister chromatids in haploid and *dmc1* mutant. However, in both contexts this number of putative sister CO is lower than expected (5 for the haploid and 10 for the diploid), if the ratio of CO/DSB would be the same as in wild-type diploids. This suggests that DSBs are less prone to be become a CO when they are repaired on the sister than when they are repaired on the homologue. CO between sister chromatids at low frequency were observed in maize (Schwartz, 1953). Such a bias that favor CO outcome when a DSB is repaired on the homologue and not on the sister has been recently proposed by a study in *S. cerevisiae* [13], suggesting that this is a general phenomenon at meiosis.

## Materials and Methods

### Plant material and growth conditions

Plants were cultivated with a 16 hours day and 8 hours night photoperiod, at 20° C. Haploid *Arabidopsis thaliana* plants were produced by crossing GEM plants [16,37] with wild-type Col-0 plants. Mutant haploids were obtained by crossing GEM with heterozygous plants for each mutation. Double and triple mutant haploids were obtained by crossing double or triple heterozygous plants by GEM, respectively. The lines used for producing mutant haploids and genotyping were previously described: At*spo11-1-3* (N646172) [57], At*rec8-3* (N836037) [49], *rad51-1* [45], *dmc1-3* (N871769) [58], *osd1-1-3* (Koncz collection, Cologne), [59]. 

### Cytology

Meiotic chromosome spreads and immunolocalizations were performed as described previously [5,38,39,42].

### Ploidy analysis

Haploid *MiMe* offspring ploidy analyses were performed by flow cytometry as described previously [60].
